# Cases and Observations, Illustrative of Renal Disease, Accompanied with the Secretion of Albuminous Urine

**Published:** 1840-10

**Authors:** 


BRITISH AND FOREIGN

### MEDICAL REVIEW,

FOR OCTOBER, 1840.

### PART FIRST.

### Analytical and Critical Reviews.

### Art. I.

1. *Cases and Observations, illustrative of Renal Disease, accompanied with the Secretion of Albuminous Urine.* By Dr. Bright. (*Guy's Hospital Reports*, No. II.—1836.)

2. *De l'Albuminurie ou Hydropisie causée par Maladie des Reins, &c.* Par le Dr. Martin-Solon, Médecin de l'Hôpital Beaujon, &c. *Avec planches coloriées.—Paris*, 1838. 8vo, pp. 480.

*On Albuminuria or Dropsy caused by diseased Kidney.* By M. Solon, m.d.

3. *Traité des Maladies des Reins et des Altérations de la Sécrétion Urinaire, &c. Avec un Atlas in Folio.* Par P. Rayer, Médecin de l'Hôpital de la Charité, &c. Tome i., pp. 625. Tome ii., pp. 620.—*Paris*, 1839-40.

*Treatise on Diseases of the Kidneys and the Morbid States of the Urinary Secretion, &c.* By P. Rayer, m.d. &c.

4. *On Granular Degeneration of the Kidneys, and its connexion with Dropsy, Inflammations, and other Diseases.* By Robert Christison, m.d. f.r.s.e., &c.—*Edinburgh*, 1839. 8vo, pp. 288.

5. *Observations on Abdominal Tumours and Intumescence; illustrated by Cases of Renal Disease.* By Dr. Bright. (*Guy's Hospital Reports*, No. VIII.—*April*, 1839.)

6. *Cases and Observations illustrative of Renal Disease, accompanied with the Secretion of Albuminous Urine. Memoir the Second.* By Dr. Bright. (*Guy's Hospital Reports*, No. X.—*April*, 1840.)

7. *Cases of Albuminous Urine, illustrative of the Efficacy of Tartar Emetic, in Combination with other Antiphlogistic Remedies, in the Acute Forms of that Disease.* By Dr. G. H. Barlow. (*Guy's Hospital Reports*, No. X.—*April*, 1840.)

We proceed to fulfil a promise made to the readers of this Journal at the close of a former article,* that upon the appearance of another Part of M. Rayer's work, we should resume the subject of renal diseases.

In the present volume, bulky as its predecessor, M. Rayer completes

* Brit. and For. Med. Rev., Vol. VIII., p. 121.

the description of inflammations of the proper tissue of the kidneys,—the classification of which affections, as well as the characters of the *simple* variety, we have already examined. Nephritis "produced by morbid poisons," it will be seen by reference to the article alluded to, stands next on the list to that variety, and with this the author accordingly opens his second campaign. Under this title are included inflammatory changes developed in the kidneys during the course of carbuncular affections, of glanders, typhoid and yellow fevers, variola and scarlatina, and as an effect of the phlebitis or absorption of pus occasionally witnessed as a complication of wounds, either accidental or resulting from surgical operations. Here, it will be perceived, is almost untrodden ground; for, although cases illustrating the effects of such morbid states on the kidneys may be found scattered through various works, yet the subject has never been methodically considered, nor has the relation of these allied cases been seized or acknowledged.

Nephritis, developed under these circumstances, commonly a mere closing phenomenon of a disease of the system at large, is invariably of the most dangerous character, is uninfluenced—unless it be for the worse—by antiphlogistic treatment, appears in carbuncular or gangrenous disorders to result directly from the same cause as the primary disease, but in typhoid fever may almost always be traced to the influence of retention of urine; and hence is in the one case a natural constituent of the general malady, in the other an accidental complication. M. Rayer exhibits proper discretion in abstaining from giving a general description of the symptoms and anatomical characters of this variety of nephritis; the number of cases on record is in fact much too small and their details too imperfectly related to justify generalization. With the latter fault, the narrative (occupying thirteen pages) of a case observed by this author himself cannot assuredly be charged. In this instance there was severe lumbar pain at the outset, followed by gangrenous inflammation of the gums, fetid salivation, gangrene of different parts of the surface, enlargement of the lymphatic glands, vomiting of black matters, bloody stools, epistaxis, &c. In addition to cutaneous eschars, to petechial extravasations in the heart, under the pleura and peritoneum, enlargement and softening of the spleen and gangrene of the stomach, there appeared marked enlargement of the kidneys with small collections of pus in their substance, ecchymosis and gangrene of the pelvis, and bloody urine in the bladder. A case of anthrax, related by Mr. Ewen,* is here transcribed: the kidneys are represented in this instance to have been "softened and disorganized."

Many writers have observed that blood is sometimes discharged with the urine in the course of hemorrhagic smallpox (*variolæ nigræ*), and in these cases the kidneys have been found engorged with dark-coloured blood and studded with ecchymoses. Under such circumstances M. Rayer has frequently detected albumen and blood-globules in the urine. These cases are, we should conceive, rather examples of passive congestion of the organs in question, coupled with altered constitution of the blood, than of actual inflammation. M. Gendrin† has, however, related a case proving that suppurative nephritis may appear as a complication of smallpox: such an occurrence must, to say the least, be singularly rare.

Intercurrent renal inflammation has been not unfrequently noticed as

* Med. Gaz., vol. xii., p. 251. † Hist. Anat. des Inflammations, t. ii., 256.

an attendant on yellow fever; among other writers, by O'Hallaran, Rochoux, and Devèze. To the information given by these writers M. Rayer adds no original facts. The converse is the case in respect of typhoid fever;—the subject has scarcely been alluded to by his predecessors, while this observer contributes some interesting illustrations of it. We have stated that nephritis ordinarily appears as an effect of retention, but it seems from the statement of M. Rayer, to arise in some instances with such promptitude that its production cannot be thus satisfactorily accounted for. The diagnosis of the affection is obscure. The stupor of typhoid patients prevents the observer from obtaining any useful information from the state of sensibility of the renal regions; and our author instances "diminished acidity or alkalescence of the urine" (the latter according to his own previous statement occurs in one only of every twenty-five cases), "and the presence of mucus-globules, sometimes of those of the blood, with the discharge of a certain quantity of albumen," as its important signs. Although, however, the diagnosis can rarely be established with certainty, and if the disease be at once developed, we are at present acquainted with no means of controlling it, yet the observations of M. Rayer are even now practically important in showing more and more clearly the absolute necessity of carefully watching the state of the bladder, and preventing accumulation of its contents in typhoid subjects. The affection of the kidneys may, instead of amounting to actual inflammation, simply consist of hyperæmia with petechial extravasation,—a condition producing notable albuminuria.

Our author's section on purulent deposition (metastatic abscesses) in the kidneys, as a result of distant phlebitis or absorption of pus, need not detain us.

The third species of renal inflammation, the arthritic, is either gouty or rheumatismal. The former is a chronic process developed around the accumulations of uric acid in the substance of the kidney, presenting themselves with tolerable frequency in subjects of gouty diathesis. The anatomical characters of this variety of disease are the same as of simple chronic renal inflammation; but it may be distinguished during life, as well by the constitution of the patient as by the acidity of, and lithic acid deposits in, the urine, while in simple nephritis that fluid is alkaline, and its sediment generally composed of amorphous pulverulent matter, of crystals of the ammoniacal phosphate of magnesia, of phosphate of lime, or of lithates. There is little novelty in M. Rayer's description of nephritic colic or other occasional effects of this form of disease. In affirming, however, that there exists a "rheumatismal nephritis" he appears to incur the responsibility of originality. He has, as he avers, ascertained that in many subjects dying of disease of the heart or pericardium in the course of rheumatism, the kidneys also are diseased. The morbid changes in these organs, whether recent or of long standing, are, we learn, of rather peculiar aspect. In the former case one or more collections of solid plastic lymph present themselves in the cortical substance, appearing externally in the form of prominent yellow patches. The corresponding portions of the capsular membrane are generally injected, the size and weight of the kidneys augmented, and their tissue occasionally studded with small collections of pus. The prominences referred to disappear in the chronic disease, and are replaced by depressions, while the effused plastic lymph assumes, with the exception of its yellow tint, the characters of solid condensed cellular membrane; the capsule is generally thickened and strongly adherent opposite the morbid depressions. In drawing attention to these forms of disease, M. Rayer makes no attempt to conceal the insufficiency of his present acquaintance with their distinguishing features either after or before death. He admits that lesions, resembling those just described as belonging to the acute stage, occur in certain forms of nephritis produced by morbid poisons,—and though the fibrinous deposits of renal apoplexy are said to be distinguishable from those referred to by their deeper yellow colour, by their being *often* streaked with black lines, and accompanied with other hemorrhagic deposits of perfectly black hue, it is easy to conceive the difficulty that must, at least occasionally, arise in the just appreciation of these nice distinctions, especially as they are not maintained to be constantly present. Further, such is the obscurity of the symptoms of this affection that M. Rayer admits his inability to assign any character by which it may, during life, be recognized with tolerable certainty. Suppose even—and this remark is an important one—that in the case of rheumatism, pain, after having successively visited several joints, appears in one of the lumbar regions, we cannot be by any means satisfied, as some authors would persuade us, that the seat of the suffering may be known to be in the muscles, if it be increased by motion of the trunk,—for M. Rayer has known, and we have ourselves made a similar observation in respect of milder renal affections, the pain in the kidneys singularly increased in nephritic colic by such motion. The presence of albumen in the urine of rheumatic patients, as frequently noted by M. Rayer, is not regarded by him as evidence of the existence of inflammation;—unless attended with pain in the testicle, albuminuria is not a sure sign of nephritis or even of renal hyperæmia. If the affection be recognized, it is clear the treatment will consist in pushing antiphlogistics further than the primary disease might require. The whole subject is well worthy of further examination, more particularly as the author states that he has known this rheumatismal affection of the kidneys prove fatal in one case, where disease of the heart or its membranes had no share in causing the patient's destruction.

But enough upon these less important varieties of renal disease: in the history of the next species of inflammation, as established by M. Rayer, we should find abundant materials to engage the reader's attention through a much longer space than the plan of this Journal will permit us to engross. Let us, however, enter as fully as possible into an examination of the remarkable disease termed albuminous nephritis by the French writer, and well known in this country as "Bright's disease."

Dr. Bright, it appears, took distinct cognizance of the chronic and advanced anatomical conditions of the disease alone; and it follows likewise, from distinct avowal in his pages, that he was dubious as to the precise nature of the connexion of the lesions he described. It is true this observer has figured a distinct example of hyperæmic enlargement of the kidney, attended during life with anasarca and a coagulable state of the urine, but so little comparative importance did he attach to this morbid change, that in the general description of the disease, in his original essay, it is not made the subject of reference, while in his second production, though the complaint is spoken of as commencing with acute symptoms, no cases are related from which the writer's opinion respecting the anatomical state of the organs, where such symptoms exist, may be gathered with precision. The attempt exhibited in the next column to trace a regular catenation of morbid changes from the mere derangement of local circulation to the most advanced disorganization must, if well founded, confer high distinction on M. Rayer. Now, that the first stage of disease exists, as described by this pathologist, seems established—the statements of others corroborate his account. And it is equally certain that we have here the anatomical evidences of intense congestion, if not of actual inflammation. The enlargement of the organ is the simple result of the stagnation of blood, and it would be not more erroneous to call an erect penis hypertrophous than, with M. Solon, thus to designate the congested kidney.* M. Solon, it is true, talks about the blood being already combined with the renal tissue in this stage of the disease, but the alleged fact that that fluid cannot be removed by washing is no proof of this,—more especially as it may be easily expressed. The characteristic feature of the next phasis, the mottled appearance produced by reddish maculæ on a yellowish ground, seen both on the external surface and in the interior of the kidney, is attributed by M. Rayer to the partial disappearance of the previous state of hyperæmia and its replacement by anæmia. M. Solon conceives, on the contrary, that the yellow tint results from more complete combination of the principles of the blood with the renal tissue, and not from a bloodless condition of the part; an opinion against which the objection already made, and obviously applicable during the first stage, now ceases to bear. In the third, the hyperæmia disappears more completely, the mottled aspect is lost, and a uniform slightly yellowish tint prevails: anæmia is now, according to M. Rayer, general. But, as M. Solon well observes, the term anæmia, which may be very correctly employed in speaking of the state of the kidneys in subjects dying of hemorrhage, is not applicable, in point of accurate description, to the discoloration now referred to. And M. Rayer himself elsewhere lends force to this observation; for he points out (p. 324) its yellow tint as actually distinguishing this condition of the renal tissue from the anæmic kidneys of certain tuberculous subjects. In the fourth phasis the previous state of discoloration still prevails, but the deposition of "Bright's granulations" marks it distinctly; the fifth seems to be a mere modification of this stage. In the sixth, the granulations commonly disappear, and differing in this from all its predecessors, this stage is frequently marked by a tendency to congestion, contraction, or atrophy on the part of the kidney. M. Solon, it will be seen, carries us a step further, and attempts to range all renal, analogous, and heterologous products as an ordinary and necessary sequence of Bright's disease,—a proceeding so utterly irreconcileable with what is known respecting those products and with the general laws of pathology, that we can only marvel at its adoption.

The anatomical characters of the affection claims our first attention, and in order to exhibit in a distinct manner the similitudes and differences in the descriptions of the four observers who have made them a subject of special study, we shall display these in a condensed form in juxta-position.

* This is a singular notion on the part of a writer of M. Solon's experience. Dr. Christison, however, has no excuse for joining, in his allusion to it, in a habit reprehensibly common both among ourselves, and among foreigners in their references to English literature, namely, in ascribing to a people at large the opinions of individuals; he fathers the present mistake upon the entire community of "French pathological writers."

BRIGHT. (1827.)

*Chronic.*

1st *Form.* The kidney loses its firmness, acquires a yellow or mottled appearance externally; the same yellow colour, slightly tinged with gray, pervades the cortical substance; the tubular is of lighter colour than natural; the size of the kidney not materially altered; there is no morbid deposit.

2d *Form.* The whole cortical part is converted into a granulated texture, with copious morbid interstitial deposit of an opaque white substance: the kidney is generally enlarged, sometimes very much so. (The granulations are rendered more apparent by maceration.)

3d *Form.* The kidney is quite rough, and scabrous to the touch externally, and is seen to rise in numerous projections of about the size of a large pin's head, of yellow red and purplish colour. The form often inclined to be lobulated, the feel hard, the texture ef semi-cartilaginous firmness: tubular portions appear drawn near to the surface of the kidney.

RAYER. (1837.)

*Acute.*

1st *Form.* Kidneys enlarged, their weight may reach 12 oz.; firm but not hard; surface of a morbid red colour and studded with small deep red points. Internally, the increased size is found to depend on the cortical substance which presents a great number of similar points, apparently the Malpighian glands injected. Tubular cones of duller red colour, and their striæ less distinct than natural; pelvis and calices injected.

2d *Form.* The enlargement persists, with slight diminution of consistence; tendency to lobulation is often observed; mottled appearance from red spots on a yellowish white ground (mixture of hyperæmia and anæmia.) On division, the cortical substance appears swollen and of pale yellowish tinge, speckled with red; the tubular of a rather bright brownish red.

*Usually Chronic, rarely Acute.*

3d *Form.* Size and weight increased as before; no mottling; cortical substance externally and on section appears of a pinkish white and slightly yellowish hue, or paler and like that of eel's flesh. Small vascular arborizations; and sometimes large white granulations resulting from deposition of plastic lymph.

4th *Form.* Size and weight as before; external surface smooth, of pale yellow colour, speckled or covered with milky white spots as large as the head of a very small pin; these are found also in the interior of the cortical substance (which is of the same pale colour as in the two previous forms), and aggregated into flocculent streaks.

*Chronic.*

5th *Form.* Rarer than the preceding; kidneys as before in point of size and weight; lobules unnaturally distinct; the external surface appears as if a vast number of "grains de semoule" were deposited under the cellular capsule of the organ.

6th *Form.* The kidney is sometimes larger but often smaller than in health, is hard and presents inequalities or mamillæ on the surface, few or no milky spots (Bright's granulations), but commonly some of these in the interior of the cortical substance. Capsular membrane almost always thickened and very adherent.

MARTIN-SOLON. (1838.)

1st *Degree or variety.* Kidney red, hypertrophous, enlarged and heavy—especially cortical substance; the tubular is also of deep red colour, but not hypertrophous; the blood combined with these tissues cannot be removed by washing; the renal substance is friable and marked with red or blackish stellate points—probably ecchymoses.

2d. Tissue, still hypertrophous, presents a yellowish striated or mottled appearance; the sulci marking the divisions of the kidney in infancy are sometimes manifest; the tubular substance is slightly hyperæmic.

3d. Kidney almost always hypertrophous, a state still depending on cortical substance; the external surface, generally smooth, sometimes presents inequalities. Surface of a pale yellow hue, something like that of the pancreas, as likewise is the substance of the kidney internally; the cortical substance appears to penetrate between the radii of the tubular; and these latter have in some measure disappeared or tend to become of pallid colour. The tissue is soft, but to a certain degree friable, though it resists laceration somewhat.

4th. The kidney presents the yellow appearance just described; and besides white pultaceous creamy particles, apparently produced by interstitial exhalation on the surface and in the substance of the organ (Bright's granulations.)

5th. Kidney, in addition to the anatomical characters of Bright's disease, contains some form of adventitious product (cysts, tubercles, carcinoma, &c.)

CHRISTISON. (1839.)

1. *Incipient Stage.* A minor degree of the second stage,—namely, of the deposition of a grayish-yellow, obscurely granular matter in the cortical structure, with or possibly without some degree of sanguineous congestion.

2. *Middle Stage.* The deposition of granular or cheese-like matter, the only important and well-established anatomical character of the morbid formation, seems at first to be, for the most part, chiefly confined to the cortical substance.

3. *Advanced Stage.* The morbid deposition gradually pervades the tubular substance.*

* Dr. Christison also believes that the following appearances "ought to be distinguished with the view of afterwards tracing their relationship. 1. Congestion of the kidneys with or without some granular deposit in their substance. 2. True granular degeneration of the cortical or tubular structure; *a*, finely granular; *b*, botryoidal. 3. Degeneration by a smooth homogeneous yellowish gray mass intermediate in consistence between that of the liver and that of the brain. 4. Disseminated tubercles. 5. Induration of semi-cartilaginous hardness. 6. Atrophy with disappearance of the proper renal structure, and with or without one of the previous morbid states. 7. Simple anæmia."

Now it must be admitted that the gradations of disease here described seem naturally and closely connected, as far as the fourth; and though it may be difficult to demonstrate the link between the yellowish discolorations, &c. and the deposition of the granular matter, the fact is no less important that such deposition does not probably occur until the tissue of the kidney has, with greater or less rapidity, passed through the previously described phases. Should a suspicion arise that accuracy has been sacrificed to zeal for systematic arrangement, this may be dismissed with the reflection that each of these forms of disease with its special characters has repeatedly been observed by writers who entertained no particular view respecting the mode of relation of the series. The sixth stage of M. Rayer, acknowledges its connexion with its predecessors by the occasional presence of the milky granulations. Such then may, in the present state of knowledge, be fairly admitted to be the mode of progress of this disease under ordinary circumstances, and when its evolution is regularly accomplished. But it must not be forgot that, in many instances, we are without any direct proof from anatomy, or collateral evidence from symptoms, that the disorder has originated in active congestion: the morbid changes appear indeed to advance so insensibly as almost to exclude the notion of an irritative process having existed in the affected organ.

In our arrangement of M. Rayer's *forms*, we have shown the presumed connexion between the anatomical lesion and the acute or chronic course of the symptoms. The acute affection has most frequently been witnessed in children as a sequence of scarlatina, especially according to M. Rayer, in certain epidemics, but also occurs in adults independently of any exanthematous disorder. In these cases it appears with the ordinary character of a febrile disease, is attended commonly with sickness and vomiting, and characterized by certain remarkable changes in the constitution of the urine and blood, and by effusion of serosity into the cellular membrane, or more rarely into the serous cavities. The urine is, at this period, always acid and voided in small quantity; it is at times, according to Dr. Christison, altogether suppressed, but there is little evidence of this in his reported cases, and of the nature of one of those (No. 1), apparently justifying the statement, serious doubts, as we shall presently see, may be very fairly entertained. The colour of the fluid is reddish or deep brown, depending on the presence of more or less blood; this is in rare cases so abundant as to be voided in small clots. We have already alluded (Vol. VIII., p. 128,) to the dissent between observers respecting the specific gravity of the urine in this affection. M. Rayer now somewhat modifies his former statement on the point by affirming that the density is *often* above and *rarely* below the healthy standard. The average of six cases, observed by himself and Dr. Bright, gives 1028; and if, as he remarks, intercurrent inflammation of other organs arise, the density increases still further. Dr. Christison's statements on this point are deficient in explicitness; for at page 34 he asserts that "the density now lies within the limits of health," while at page 48 we find "a moderate reduction" of specific gravity recorded among the pathognomonic characters of the incipient stage. M. Solon has on his side fallen into the serious error of omitting to distinguish, in speaking of the density of the fluid, the periods of the disease at which the observation is made. There can be no doubt that the specific gravity is comparatively high at the outset of the complaint, a fact explained by Rayer by the simple consideration that the ratio of the solid to the aqueous constituents of the urine is at this period scarcely affected. Dr. Christison, who maintains that the proportional quantity of solids is already lessened, refers the high average density to the adventitious albumen which is present, strengthening this opinion by the allegation that "if the fluid be filtered after coagulation the density falls by four, five, or even seven units." But here is a most fallacious argument; for the process just mentioned separate, not only the albumen, but also always a portion of urea, and frequently a large share of mucus or blood globules, of lithic acid, and lithate of ammonia, (*vid.* Vol. VIII., pp. 135-6.) The old mode of clarifying coffee might have reminded Dr. Christison of this fact. We are therefore disposed to agree on this point with M. Rayer. Every one will of course grant Dr. Christison that, as the quantity of urine discharged is commonly below the normal average, the total amount of solids excreted therewith in the twenty-four hours falls below the healthy standard; but the estimate, that it falls to one fourth or one sixth of the ordinary mean is not to be confided in, as the error just referred to must have influenced this calculation also.

Examined under the microscope, the urine is seen to contain blood-globules in numbers, occasionally mucus-globules, and always lamellæ of epithelium: at this stage crystals of uric acid are rarely observed. The sanguinolent appearance may obtain for two or three days or more, and sometimes recurs after disappearance; the degree of bloody impregnation varies from time to time, as that of the albuminous, and the abundance of the latter is not at all in the direct ratio of the former. The quantity of albumen discharged varies not only in different patients but in the same subject from day to day, nay, even from hour to hour. Dr. Christison affirms that it is always abundant during this stage, but may suddenly disappear temporarily: Rayer has "often" found the pale urine of the chronic malady "much more highly albuminous" than the deep red fluid voided in the acute stage.

The renal regions are commonly the seat of dull, rarely of acute pain. Dr. Christison speaks of frequent desire to pass urine, accompanied with difficulty or pain in the act, as existing at this period: M. Rayer affirms that these phenomena are never present except there be coexisting disease of the bladder, or fibrinous concretions of large size present themselves at the orifice of the urethra. The retraction of the testicle and pain in the direction of the ureters, sometimes observed in simple nephritis, do not appear to exist in this affection. Scarcely have the morbid changes of the urine been established, when anasarca supervenes, ordinarily commencing by puffiness of the eyelids or face, in other cases originating in the limbs, and characterized by tenseness of the skin and the absence of pitting under pressure.

To the frequency of buffiness in blood, drawn at this period of the disease, we have the testimony of all observers who have written on the subject. The specific gravity of the serum diminishes from the decreased proportion of its albumen, and may fall from 1030, the normal mean, to 1022, or even 1020 or 1019. Rayer states that the natural state may return a few days after venesection, provided that operation have rendered the urine less albuminous than it had previously been. The serum is sometimes lactescent from admixture with fatty matter removable with sulphuric æther. According to Dr. Christison, "the presence of a large quantity of urea" in this fluid may be ascertained during the present stage, provided the amount of urine have not been considerably increased by incidental causes beyond what constitutes the common average at this period. MM. Rayer and Guibourt sought unsuccessfully for albumen in two instances during the first stage. Negative evidence will not in circumstances like these, more especially as M. Rayer does not mention the quantity of urine daily discharged, counterbalance the positive assertion of Dr. Christison. But his proposition appears open to attack on other grounds. In truth, on turning to Dr. Christison's collection of cases we find not a single one conclusive of the alleged fact. Cases i. and xx. are probably those to which the author would direct our attention; but that the former was a case of Bright's disease at all may be doubted; and as death did not occur in the latter, the precise state of the kidneys can only be matter of conjecture; but that it was far from being exactly such as the narrator of the case would infer appears from the following considerations :—The subject whose history is therein given had had anasarca twice previously, twenty years and five years before he presented himself at the Edinburgh infirmary. Granted that he had in the interval been in apparent possession of good health, and that his last attack supervened with acute symptoms ; where is the proof that the man had not been voiding albuminous urine for months, for years, in a word, that the disease had not in the interim been following a latent course ? Does not Dr. Christison here fall into the very error against which he himself, even more emphatically than his coadjutors in the investigation of this disease, warns others ? Does he not totally forget the existence of his own 28th, 29th, and 67th pages, where he says there are many cases where, although the disorder may *appear* to have begun as an acute affection, traces will be found of its having existed for several months before in a chronic form ; [where is this more likely than in a case where there had been, at least, two distinct attacks previously ?] and further relates that the kidneys of a stout, muscular, and healthy woman, who had been killed in a squabble, were found " very far advanced in granular disorganization." Had this woman been the subject of an acute intercurrent attack before death and fallen under the notice of Dr. Christison, her blood would have furnished an excellent example of impregnation with urea " in the incipient stage of the affection." But, again, Dr. Christison's mode of satisfying himself of the presence of urea, in some cases, may have its share in inducing the discrepancy of opinion referred to. It appears, that he considers effervescence with evolution of an urinous odour by the action of nitric acid on the alcoholic extract of the solids of the serum, a satisfactory proof of the presence of urea. Now, Lecanu,* and Brett, and Bird,† have shown that the peculiar odour in question is evolved when a certain extractive matter of the blood, wholly distinct in nature and properties from urea, is thus treated. Besides, MM. Guibourt and Rayer have ascertained that small solid masses simulating the nitrate of urea in sensible properties, may be obtained by the reaction of nitric acid on certain alcoholic extracts of the serum. The proportion of fibrine now varies, according to Dr. Christison, from 82 to 30 parts in 10,000—a tolerable proof of the insignificance of its ratio as an evidence of the presence of the disease : even in health the quantity of fibrine is subject to very extensive variation. Dr. Christison believes that the ratio is, in Bright's disease, " regulated by the amount of buffiness of the blood ;" but M. Rayer well reminds us that, as Denis has shown, the quantity of fibrine cannot in other diseases be calculated by that of the buff. The proportion of hematosine is stated by Dr. Christison to be undecreased in amount during this stage. His calculation really refers to the globules and not to the hematosine ; he himself appears to consider them the same thing.

The disease may terminate by recovery, by death, or by passing into the chronic forms. The former termination is announced by abundant sweating, by marked increase of discharge from the kidneys, with restoration of the natural characters of that discharge, and disappearance of the dropsical effusion. When the disease proves destructive to life, the fatal issue is generally preceded by cerebral symptoms or by thoracic in-

* Brit. and For. Med. Rev., Vol. VI p. 433.
† Med. Gaz., vol. xii., pp. 494, 567, 805.

flammation. If the complaint subsides into the chronic state, the patient may recover the general appearance of health, and no sign of morbid character be present except albuminous impregnation, a point, practically speaking, of the greatest importance. This state may have continued for a variable period, when a new attack of dropsy occurs, the disease assuming the aspect of an acute disorder. A fact upon which M. Rayer insists is, that in individuals of a habit rendered cachectic by disease, or by default of healthy nourishment, the disease may wear a chronic character *ab initio,* from the absence of marked symptoms of reaction, and yet the kidneys display on inspection the anatomical characters, though in an ill-marked form, of the acute complaint. And it is also a well-founded remark of this observer that with each recurrence there is a stronger tendency exhibited to the chronic character, though, as we have just had occasion to hint, this is a rule not without its exceptions.

In the chronic, as in the acute disease, the state of the urine and of the blood, and the presence of serous effusion, furnish its principal signs: there is rarely distinct pain or tenderness under pressure in the renal regions. The urine is sometimes voided more frequently than in health. Dr. Christison dwells forcibly upon the diagnostic importance of the patient's "being awakened once or oftener in the night-time by the necessity of passing urine;" an evident proof how completely Dr. Christison's mind is engrossed by this affection, for there is probably not a single irritative state of any part of the *lower* portion of the urinary passages especially that is not productive of similar discomfort. The same writer observes, that the quantity of urine is often very little reduced below the standard of health, frequently it rather exceeds than falls short of it, but if an acute attack supervene, or if the chronic disorganization "has been allowed to go on to an excessive extent, without the case being cut short, as more usually happens, by some fatal secondary affection," it may diminish to almost total suppression: in a case of the latter kind, "the quantity, for nine days before death, did not exceed an ounce." This fluid is now commonly slightly acid, occasionally neutral or alkaline. M. Solon remarks that alkalescence cannot depend on the presence of ammoniacal carbonate, on account of the freedom from fetid odour, or from effervescence under nitric acid, and ascribes it to the sodaic salts of the serum, which find their way into the urine along with its albumen. Were this, however, a perfectly correct explanation, we should expect to find alkalescence in the direct ratio of albuminous impregnation, which is far from being the fact. The density of the urine is now invariably low, and may fall to 1004, an effect produced by diminution of its solid constituents. It is not known in what proportion this diminution affects the urea and salts respectively: M. Solon has pointed out the deficiency of calcareous salts. The fluid is pale, with scarcely any urinous odour; occasionally turbid, contains lamellæ of epithelium, and, as its chief characteristic, a variable quantity of albumen. Its sediment sometimes contains mucus-globules or blood-globules and small crystals of lithic acid; in very rare cases some pulverulent lithates are discoverable; the phosphates also are present in very feeble proportion. The slight muddiness or turbidity occasionally observed is, according to Dr. Christison, "probably owing to modified vesical mucus," but it may also depend on the suspension of fatty matter removable with sulphuric æther, as shown by

Rayer. Respecting the relative amount of albuminous impregnation in the two stages of the disease, authors vary. Dr. Osborne relates that the extent of disease discovered after death has been, according to his experience, invariably in proportion to the degree of coagulability. Rayer has found the "quantity of coagulum often greater in the acute than in the chronic disease," the whole sample of urine acted upon sometimes forming into a mass; in others, slight opalescence only being produced. It appears clearly from his chapter on the "progress of the disease," that M. Solon holds an increasing proportion of albumen to be indicative of advancing disorganization. Dr. Christison's "observation leads to the inference that the albumen abounds most in the early stage, decreases towards the advanced stage, and when abundant in the latter period, is so *incidentally from the supervention of fresh reaction.*" We confess ourselves unable to account for the difference of opinion here exhibited; unless the proposition printed in italics afford the clue thereto.

Meanwhile, what is the state of the blood? As may be anticipated from what has just been said respecting the urine, contradictory notions are held on this point. If we credit Dr. Christison, "the density and solid contents of the serum, previously much reduced, gradually return to the healthy standard, or even exceed it;" in the middle stage the density is said to be about 1024; in the advanced it may be so high as 1031. The proportion of the solid constituents of the serum is on the contrary, according to Rayer, remarkably lowered to such a degree that he has found the specific gravity fallen to 1029, 1019, and even 1013. It will be observed, that in both instances the density of the serum is inversely as the different proportional quantity of albumen presumed by each of these writers to be passed with the urine; each of these has at least the merit of consistency. On this subject M. Rayer remarks, "*if* by beginning of the disease Dr. Christison understand the end of the first, or the course of the second month of the complaint, the diminution in the density of the serum is beyond a doubt sometimes very considerable; *if* by middle stage he designate cases in which, in consequence of amendment in the patient's state, a decreasing quantity of albumen is discharged with the urine, this statement is still correct. But the diminution of albumen in the urine and augmented density of the serum stated by Dr. Christison to be the ordinary characteristics of the final stage, appear to me to be of completely exceptional occurrence." (vol. ii., p. 122.) Your *if* would then fain be a peacemaker among pathological wranglers; but in this instance, at least, its pacific efforts fail, as Dr. Christison does not distinctly state anything of the kind alluded to. Nevertheless, there may be some share of justness in M. Rayer's supposition, for, judging from Dr. Christison's published cases, it may be questioned whether this practitioner has had an opportunity of observing a single indisputable example of Bright's disease in the truly acute stage; an idea which receives corroboration from the fact that he himself places "granular deposition" among the anatomical characters of the very earliest stage. Again, these observers differ: the Parisian writer has found the lactescent appearance of the serum now more marked, the Scotch less so, than in the outset of the disease. They agree, however, in stating the quantity of cruor to be commonly diminished and that of serum increased; and coincide in considering the proportion of globules diminished: M. Rayer has determined the latter point by microscopical examination; Dr. Christison ascertained by chemical analysis that the ratio may sink to less than a third of the healthy average. The fibrine is now, according to the latter observer, most commonly natural in amount. But here the uniformity of opinion ceases. According to Dr. Christison, the urea "*frequently* disappears from the serum of the blood as the disease advances;" while M. Rayer affirms that this proximate principle may be *more frequently* detected in the chronic than in the acute stage. The Edinburgh Professor modifies the above opinion so materially in the very sentence in which it is announced, as to involve at the least, a contradiction in terms: "in the most advanced stage," he says, "the urea *commonly* reappears, and it is sometimes present towards the close in larger proportion than ever." The variation depends, it is alleged, on the varying quantity of urea excreted by the kidneys; whenever this is materially reduced, the principle in question may be distinctly found in the blood.

The characters of the anasarca which forms an almost invariable attendant on this stage of the affection are sufficiently well known. But respecting the importance of anasarca as a symptom there is some difference of opinion. The French describers of Bright's disease, struck with its extreme frequency, rank it among the regular symptoms of the affection; Dr. Christison, influenced by its occasional absence, lowers it in importance to that of a mere secondary affection. If the arrangement of Dr. Christison be more scientific, (this, however, may be doubted, for we are not aware that the signification of the phrase "secondary affection" has ever been defined with precision,) that of the French authors is more practically useful, inasmuch as less danger is to be apprehended from slight exaggeration of the frequency of the condition in question, than from underrating the constancy of its occurrence. Dr. Christison upbraids the continental writers on the subject with "incorrectly considering anasarca an essential character of the disease;" but the accusation is a groundless one: M. Solon (p. 267) recognizes the occasional absence of anasarca as an ascertained fact; M. Rayer has figured the kidneys of individuals who had not suffered from this symptom, and M. Forget published a similar case. Whatever be the term applied to indicate the connexion of anasarca and the renal lesion, the important point to remember is, that the former is scarcely a less frequent attendant on the latter than, for example, cough on phthisis.

The volumes of MM. Rayer, Solon, and Christison contain numerous reports of cases in justification of the general statements broached by their authors. We have much satisfaction in perceiving in those of the latter writer, a very distinct improvement upon the ordinary style of records of the kind published in this country; and though numerous defects both of matter and manner might easily be pointed out, we shall, in compliment to the general superiority of the whole, suffer these to pass without particular notice. M. Rayer's cases are divided into two grand sections: the first exhibiting the characters of the uncomplicated disease; the second illustrating its multitudinous relations of cause and effect to certain organic and functional maladies. In his commentary on these cases, the author examines some of the most important questions connected with the general pathology of the disease. Opportunities of ascertaining the condition of the kidneys in the truly acute stage have rarely occurred, and from the statements already laid before the reader, it follows that in the present state of knowledge there can be little surety in many instances respecting the stage of the disease from the evidence of symptoms. Besides, in respect of other renal affections, the diagnosis of the early stage of Bright's disease is anything but satisfactorily established. In proof of this, it is sufficient for the present to state that two cases, reported by M. Solon as distinct examples of the incipient affection, are regarded by M. Rayer as cases of inflammation of the pelvis, of the kidney and bladder: that Dr. Christison's case i., put forward as exhibiting "a characteristic example of the early stage of granular disorganization," is by the same French writers esteemed a case of *simple* nephritis attended with typhoid symptoms; and again, the patient of the Edinburgh author's case xix., supposed by him to have been the subject of the early stage of Bright's disease, is by M. Rayer maintained to have suffered from simple hematuria. In many cases of acute dropsy with coagulable urine, and this more especially where renal pain coexists, the kidneys are probably the seat of such congestion as would ultimately lead to the special disorganization, but it would be no easy task at present to demonstrate this, as the following paragraph will show. In the infancy of our acquaintance with a disease, we can rarely acquire scientific certainty of diagnosis unless with the assistance of the scalpel. M. Solon adds one distinct example, with the dissection, of the incipient stage of the disease to that published by Dr. Bright; M. Rayer three or perhaps four of the same kind.

We pass on to the section of M. Rayer's commentary, in which the relation of this disease to other morbid states of the urinary organs are the subject of enquiry. The result of his experience on this matter is extremely important. "I have already," he says, "had occasion to point out frequently the striking analogies between simple and albuminous nephritis. The action of cold and damp produces both diseases. In the acute stage (with the exception of deposition of pus, which has either not been observed at all, or at least very seldom, in Bright's disease,) the anatomical characters are identically the same—the injection of the kidneys, their increased size, yellow discoloration, &c., are all appearances common to both maladies. In the most advanced chronic stage, the lesions are so precisely similar, that were it not for several circumstances connected with the progress of these affections, the absence or presence of dropsy, and the constant or only occasional presence of albumen in the urine, it would be impossible to distinguish them from each other." But, adds this author, there are striking points of distinction also between these diseases, the only one cited being the marked influence of morbid states of the remainder of the urinary passages or simple nephritis, while these are presumed to be without, or at least to have very slight influence on the albuminous species. M. Solon considers simple nephritis distinguishable from Bright's disease by the absence of œdema and the presence of renal pain, nausea, and vomiting. But it must be remembered that Bright's disease, as admitted by M. Solon himself, is not attended in all cases with anasarca, and the symptoms, mentioned as peculiar to simple nephritis, decidedly occur in the so-called albuminous variety. Dr. Christison's experience does not allow him to speak decidedly on the subject, "as simple nephritis is an extremely rare disease in Edinburgh." In the accuracy of the latter statement we see no reason for coinciding; it is a notorious fact, that some years since, before renal pathology had excited the attention it has of late done, the persuasion of the rarity of nephritis was universal. We have ourselves heard most eminent Parisian pathologists affirm that they had scarcely ever seen an example of nephritis—men who now in the short space of a session find abundant materials for lectures on the disease among the patients of a single ward. And again, how Dr. Christison managed to draw the inference from Rayer's works that that pathologist maintains atrophy to be peculiar to and distinctive of the *simple* chronic nephritis, we are at a loss to understand. So far is this from being the truth, that Rayer has actually figured five examples of atrophy as a dependence upon the disease described by Dr. Bright.

The reader is most probably acquainted with the case published by the late Dr. Gregory, (Ed. Med. and Surg. Jour., vol. xxxvi., p. 359,) as exemplifying the production of Bright's disease under the influence of lithotomy: the kidneys are represented to have been mottled and to have contained internally a considerable quantity of granular matter. This is regarded by M. Rayer as an example of simple nephritis, an affection of most common occurrence under the circumstances described by Dr. Gregory: the mottled appearance is quite as frequent in the latter form of disease as in "albuminous nephritis;" and Rayer is of opinion that the granular matter must in reality have consisted of pus or plastic lymph. The case furnishes an excellent illustration of the necessity of minuteness and accuracy of detail, particularly with respect to subjects of novelty. Albuminuria after lithotomy by no means proves the existence of Bright's disease; Rayer has repeatedly discovered albumen and blood-globules in the urine of subjects suffering from *simple* nephritis and cystitis after that operation.

The correctness of Dr. Christison's diagnosis in several of his reported cases is contested, and not without apparent foundation, by the French pathologist. We have already mentioned that the first case in his work is regarded by M. Rayer as an example of simple nephritis with typhoid symptoms; the truth is, that renal pathology is so little advanced that it would be presumptuous in us to attempt to decide between these discordant writers, at least in respect of the present question, yet we may be permitted to say that to our minds there is an *à priori* force in M. Rayer's objection, from the fact, that it is—or, at least was formerly,—too much the practice of Edinburgh, to set down as typhoid fever every inflammatory affection attended with typhoid symptoms. Dr. Christison's third and eighteenth cases, we fully agree with Rayer in considering examples of common inflammation affecting the bladder and kidneys, and wholly different from Bright's disease. And in his remark that Dr. Christison appears much too prone to infer the presence of granular disease from the occurrence of albuminuria, M. Rayer will, we apprehend, be joined by every one who impartially studies the work of the former. Again, the Edinburgh writer's nineteenth case refers to a subject who had abundant hemorrhage from the urinary passages followed by albuminuria, and is by the narrator considered an example of the special affection; while by M. Rayer it is esteemed an unequivocal instance of essential hæmaturia.

Dr. Christison considers (apparently on the authority of Martin-Solon) that " in hæmaturia without granular disease, the urine ceases to contain albumen, as soon as it ceases to present the colour of blood :" but his antagonist affirms that he has frequently found the urine in hæmaturia continue albuminous for some time after it had ceased to contain blood-globules, either in suspension or in its sediment.

M. Rayer considers diseases of the bladder, prostate, and urethra without influence on the development of " albuminous nephritis," and has found that the latter affection rarely induces inflammation in the pelvis and ureters, and still more rarely in the bladder. He seems disposed, however, to admit that diabetes may have some influence in its production. Certain it is, that saccharine diabetes not very uncommonly passes into dropsy with albuminous urine ; but, though Dr. Bardsley declares (Cyclo. of Prac. Med., vol. i., p. 543,) he has observed the lesions of Bright's disease in subjects dying with diabetes, this is not a fact generally recognized, and M. Rayer reports a case (somewhat contradictory of his opinion on this subject stated in our former notice) serving to show that, as has been alleged by others, the transformation of saccharine into albuminous urine may be a sign of amendment ; if such be its import, it is difficult to suppose it an effect of the development of Bright's disease.

The attention of the observers of this disease has been drawn to its frequent accompaniment by morbid states of the heart. Dr. Bright having found sixty-seven cases of cardiac affection (hypertrophy with or without valvular disease) among one hundred of the renal disorganization, infers, without making any enquiry into their relative priority, that the latter invariably acted as the cause of the former : he further undertakes to explain their mode of connexion—" either the altered quality of the blood affords irregular and unwonted stimulus to the organ immediately, or it so affects the minute and capillary circulation as to render greater action necessary to force the blood through the distant subdivisions of the vascular system"—hence, it is presumed, the occurrence of hypertrophy : we are not informed how Dr. Bright has ascertained that morbid states of the blood produce necessarily the alleged effects, and it is obvious that affections of the valves are in this theory left altogether unexplained. Dr. Christison does not express himself very clearly or decisively as to the relation of the two states of disease, but does not adopt the curious conclusion that the cardiac are always secondary to the renal changes. M. Rayer infers from his own experience that in a very few cases indeed are the lesions of the heart an effect of those of the kidneys : he believes that Bright's disease is, on the contrary, very frequently produced by that of the central organ of the circulation, and has observed slight albuminuria, in subjects affected with hypertrophy or valvular lesions, gradually become intensely marked and attended with the most distinct evidences of the special renal affection. He adds that albuminuria, existing in subjects labouring under disease of the heart or large vessels, with or without dropsy, is not pathognomic of Bright's disease ; that, according to his experience, the urine of such patients may contain albumen either from the kidneys becoming simply hyperæmic, or without their undergoing any apparent change whatsoever,—thus confirming the original statement of Dr. Darwall. He has even observed two cases of chronic pericarditis attended with discharge of albuminous urine towards the close of life, the kidneys appearing on dissection simply congested ; and affirms that the derangement of the circulation consequent on endocarditis may cause the escape of albumen with the urine in cases where no renal lesion of any kind exists.* In these instances, the high specific gravity of the fluid, and the larger proportion of lithates and of urea therein contained, are pointed out as peculiarities which distinguish it from the excretion in marked chronic cases of the renal disease.

All these writers coincide in their statements respecting the extreme frequency of bronchitis as a secondary disease. M. Rayer observed it in seven eighths of his cases. It is sometimes attended with very copious secretion (bronchorrhœa), and may coexist with vesicular emphysema, and lead to pulmonary œdema, or, it is affirmed, lobular pneumonia. Dr. Bright found "recent or old traces" of pneumonia in one ninth of his fatal cases; Dr. Christison has only observed this complication in two instances ; M. Rayer in, at least, one twelfth of his patients. Reviewing the experience of the four writers, it appears that pleurisy—exclusive of cases in which it evidently depended on pulmonary turbercles or pneumonia—is an affection of rare occurrence in the course of Bright's disease, although the original describer of the latter affection has maintained a somewhat different opinion. The existence of old pleuritic adhesions does not appear to have been more common than in subjects dying of all diseases indiscriminately, with the exception of phthisis. Both Dr. Bright and M. Rayer point out the extreme frequency of pulmonary œdema : both have observed it in one third of fatal cases. But their experience differs respecting the connexion of phthisis and the renal disease. Dr. Bright having rarely found them coexistent, and having noticed that in some cases tuberculous disorganization appeared "to have made a certain inroad upon the upper lobes and then to have sunk into a state of quiescence or entirely subsided," is inclined to the persuasion "that so far from these diseases being *associated*, the condition of the body in this form of renal disease is unfavorable to the existence of phthisis." M. Martin Solon adopts a similar notion. Dr. Christison, on the contrary, maintains that the renal affection sometimes occurs as a secondary disease in the course of phthisis ; and M. Rayer advocates the same opinion, on the ground of his having observed every form of the disease—in many instances successively developed in the hospital—coexisting with pulmonary tuberculization, manifestly of prior development. He has also observed a certain number of cases in which pulmonary consumption appears to have arisen from the deterioration of constitution induced by Bright's disease.

"With regard to the liver and abdominal viscera generally," remarks Dr. Bright, "as compared with the heart and lungs, a very great immunity from structural disease is to be observed." In the tabular view of one hundred cases drawn up by this author, the liver is stated to have been healthy in

* As mentioned in our previous article, Dr. Christison in his pleadings for the diagnostic importance of albuminuria, throws out doubts of the efficient information of the observers who professed that cardiac disease might induce this symptom. He would himself, we doubt not, be now disinclined to push this argument, as Dr. Carswell has meanwhile published an indubitable example of the fact in the Lancet; and M. Rayer reports at length the details of a similar case, in a chapter headed " Albuminous Nephritis similated by Disease of the Heart."

forty cases, and in thirty-two others there was merely a mottled appearance, arising probably from disturbed circulation in articulo mortis: in eighteen only were there marks of confirmed disease. Now, as the subjects of these cases were, in the great majority of instances, anything but sober persons, we have here an argument of no mean force against the prevalent conventionalism respecting the direct influence of intemperance on the liver—an influence, the amount of which has, beyond the shadow of a doubt, been monstrously exaggerated.* This result is the more important, as Dr. Christison talks of conjunction of hepatic and renal disease being easily understood, *because* both are among "the infirmities of the *constitution of intemperance*." All species of lesion appear to be discovered indifferently in the liver in these cases; it occasionally contains granular matter, assimilated both by MM. Rayer and Christison to that developed in the kidneys.

The lesions observed in the other abdominal viscera and in the intestinal canal, need not delay us,—a word, however, on one of the effects of these, diarrhœa. Dr. Christison, speaking of the frequency of this symptom among the Edinburgh sufferers from Bright's disease, wishes to impress his readers with the belief that diarrhæa is comparatively a rare secondary affection in other cities where "*the habitudes* of granular disease" have been made the subject of observation. He is informed by Dr. Bright that it "has never particularly attracted his notice in London," and assured by MM. Andral and Louis, that in Paris affections of the bowels are not common. The alleged information from Dr. Bright is not easily reconcilable with the distinct written statement by this writer that "diarrhœa has carried off several patients," (Guy's Reports, No. ii., p. 339;) and as regards Paris, the experience of M. Rayer must be admitted to be more decisive on a question of the present kind than even that of the eminent persons just named; (it is besides not treating them fairly to take the statement here attributed to them *au pied de la lettre*, as misapprehension may easily arise in the verbal transmission of opinion;) now, M. Rayer has observed diarrhœa in "upwards of half his patients"—a diarrhœa remarkable for its intractability, and for its never lessening, no matter how violent it becomes, the amount of dropsical effusion:—far from this, the latter seems sometimes to increase *pari passu* with the former.

"Drowsiness and torpor are common symptoms throughout the whole disease from first to last," and so frequent are affections of the head, "more or less allied to apoplexy," of which death by coma seems the natural termination, unless life be cut short by some other incidental cause, that "it appears not quite correct to consider these affections as secondary." Does Dr. Christison here mean to affirm that such affections are more common than anasarca, which he unhesitatingly ranks with this category? The mode of progression of the torpid state which merges in

* The palmy days of liver pathology are happily gone by—yet how many victims do we still see salivated almost unto the death for "obstinate hepatic inflammation," assuming the curious shape of pleurisy—how many tuberculous subjects with wandering pleuritic pains are assured that "congestion about the liver" is the only obstacle to their enjoyment of health—how often is gastric cancer modified into "mischief going on in the liver"—and how easily might instances be multiplied in which this *favoured organ* forms the easy refuge of stolid cupidity.

irrecoverable coma, and proves the immediate cause of death in many cases, is sufficiently well known. Dr. Christison, however, in affirming without qualification that "this secondary affection may occur in the very earliest stage," broaches an opinion of debatable accuracy. At least, M. Rayer has found this of singularly rare occurrence, except in cases where the disease follows scarlatina. Dr. C. has attentively studied the relation of the state of the urinary secretion to the cerebral symptoms, and though he admits the general correctness of the common belief respecting the dependence of the latter on suppression or extreme diminution of the former, yet he shows clearly that this is not an invariable fact: "I have known," he observes, "coma form and prove speedily fatal, when thirty ounces of urine were discharged daily up to the time of death; and in case No. 3, the patient passed no more than two ounces of light urine daily for nine days before death, yet he remained sensible to the very last moment of his existence." He has also found that coma is not necessarily connected with the extent or increase of dropsical effusion. In many cases terminating fatally in this manner, the brain and meninges, it is affirmed, present no trace of lesion—a position which requires the confirmation of numerous and very minutely detailed cases before its precise truth can be admitted.* Dr. Osborne speaks of arachnitis as a common species of disease under these circumstances; and Rayer of sub-arachnoid serous infiltration as the lesion most commonly discovered, along with occasional superabundance of fluid in the ventricles: from the fluid Dr. Barlow has obtained urea. Hemorrhage into the substance of the brain, into the ventricles, or the cavity of the arachnoid, have been noted by Dr. Bright: but, on the whole, lesions of this latter kind are so exceedingly rare that M. Rayer is perhaps justified in doubting that there is any real dependence between the two states. The same must *à fortiori* be said of the "morbid tumours" discovered in the brain of one of Dr. Christison's patients, but respecting the nature of which "morbid tumours" the details, or rather the want of details, given by the author scarcely permit even a guess.

M. Rayer has observed several examples of Bright's disease as the apparent effect of pregnancy. Some of these cases were remarkable for the facility with which the disease yielded after delivery to very simple treatment. In certain instances where the disease preceded, or was developed at an early period of pregnancy, it evidently interfered with the evolution of the ovum, and in cases published both by M. Martin-Solon and himself led to fatal abortion.

A very lengthy chapter is contributed by M. Rayer on the connexion of "albuminous nephritis" and cutaneous diseases, of which the portion referring to scarlatina is alone really interesting. And in this, rendered heavy by needless prolixity, little is added to previous knowledge; even upon the most debateable point connected with the subject—the anatomical condition of the kidneys in patients suffering after that exanthema from anasarca and albuminuria—it supplies no original information: the latter omission appears to have arisen from a fortunate cause, the mildness of the affection at Paris. Yet it is fair to add that, by collecting the facts

* Of course we regard as mere non-entities cases in which the reader is obliged to content himself with the laconic statement—"brain healthy."

scattered through periodical and other works, M. Rayer has placed the reality of the occurrence of the successive stages of Bright's disease, under these circumstances, in a very clear point of view. The existence of dropsy, fever, and the presence of blood and albumen in the urine, which fluid is diminished in quantity and of low specific gravity, are the only safe signs of Bright's disease under these circumstances. Dropsy succeeding scarlatina by no means positively announces renal disorder: besides the possibility of its depending on disease of the heart or great vessels, general dropsy, unattended with coagulability of the urine, has been known to follow scarlatina, and nothing authorizes us in ascribing such affection to a lesion of the kidneys—a fact adverted to by Dr. Christison also in the same strain.

The experience of M. Rayer confirms the opinions of Drs. Hamilton and Christison respecting the influence of a scrofulous habit on the development of the disease. A case is related by the French writer, in which the urine, though strongly albuminous, contained a large proportion of lithic salts: either the patient, in this instance, was affected with renal disease or not, (she left the hospital soon after her admission;) but on either supposition, the constitution of her urine was remarkable: if she were not thus affected, by its abundant impregnation with albumen; if she were, by its full provision of urates. M. Rayer fancies that in this, and in some other similar cases, the proportion of lithates was influenced by the scrofulous habit of the patient.

From an examination of the cases published by Blackhall, Scudamore, and Bright, exemplifying the occurrence of albuminuria in gout, M. Rayer concludes that in some cases this phenomenon depends on the coexistence of Bright's disease, that in others its cause is unknown, but may possibly be "concomitant disease of the heart or a modification of diabetes."

The duration of the chronic form of Bright's disease is exceedingly variable, and indeed defies exact calculation. In the first place, we can rarely feel satisfied of the accuracy of patients in their assurance that these symptoms commenced at this or that period—not to speak of the uncertainty of such commencement as a sign of the real outbreak of the affection. Nor, even after the sufferer has placed himself under medical care, can the ulterior durarion of the disease be predicted with even tolerable certainty; if the presence of albumen in the urine have been ascertained, this throws no light on the period when dropsy may supervene; nor does the establishment of anasarca make the problem of ulterior duration of easier solution.

Authors appear to have established, much to their own satisfaction, the etiology of the disease. The exposure of the body to sudden changes of temperature, and especially to the simultaneous action of cold and damp, is set forth as the most common cause of the disorder, and as being particularly evident after scarlatina. If, however, this be the fact in acute cases—and we are not disposed to contest this—it may be doubted in respect of the chronic order. We have not only no hesitation in affirming with Dr. Christison that, "since in the generality of cases the disorder establishes itself silently and very gradually, its exciting cause must obviously prove for the most part inappreciable," but we cannot help feeling persuaded that had he observed the caution which he well states to be requisite, in taking for granted the reference often made by patients of their illnesses to cold, he would be disposed to admit, even more fully than he has done, our utter ignorance of the exciting causes of the disease. However, quasi-statistical facts are adduced by M. Rayer and others to support the current opinion;—he enumerates a host of trades, the followers of which, he alleges, are more prone to the disease than other persons. But in the first place, he seems to have collected under this head of trades, peculiarly exposing their followers to cold and damp, the most numerously peopled artisanships of Paris;—if they are stronger in numerical force, of course in the natural course of things they should furnish the largest quota of the disease in question

Dr. Christison finds "the constitution of intemperance" a most important predisposing cause of the disease, and makes a nice distinction of the spirit-bibbers into those who are habitual drunkards, and those who constantly indulge to a large amount, yet never enjoy themselves so fully as to lose their perpendicular in consequence. But where is the proof that the persons belonging to either of these distinguished classes of society furnish the chief share of patients labouring under this renal disease? Has Dr. Christison, after carefully ascertaining the proportion of drunkards to sober persons supplying the inmates of the Edinburgh Infirmary, then compared the amount of renal disease furnished by each and found the drunkards in a large majority? He dreams of nothing of the kind—and hence his inference on the subject is, to say the least, utterly valueless in a scientific point of view, more especially as, unfortunately for the moral state of the lower orders of the town population of these islands, there are few among them who would not take rank in one or other of Dr. Christison's divisions. The curious in hypothesis will find much more to gratify their propensity in the volumes before us: but we believe we shall have the sager portion of the profession with us when we affirm that nothing of an accurate kind is known on the subject—and that so it must remain until it be studied on very different principles from those followed by the ordinary constructors of etiological chapters.

The diagnosis of the disease next claims our attention. On a former occasion we entered very fully into the existing state of knowledge respecting the causes of albuminuria, and showed from the experience of the most eminent observers the extreme error of the notion—unfortunately even still too prevalent—that the mere presence of albumen in the renal secretion announces special disease of the kidneys. We shall now proceed to the examination of another point, namely, whether the amount or persistence of this impregnation is characteristic of this particular disorder, and what other qualities of that fluid may be thus characteristic. But first we must advert to a portion of Dr. Bright's most recent publication intimately connected with this subject. The chief motive of Dr. Bright in favouring the profession with this new communication was "to furnish himself with an opportunity of explaining his views on one important point connected with *the* disease attending albuminous urine, in reference to which he has been singularly misunderstood." The presumed misconception under which Dr. Bright apprehends he has suffered is, that he maintains the occurrence of albuminous urine to be always and necessarily connected with "*that organic disease* which in its various shapes and modifications has been so fully described." The learned writer then proceeds to quote from his previous writings passages showing that he conceived the functional, preceding the organic changes, to be productive in some cases of the morbid condition of the urine in question. For our parts, we never entertained the opinion respecting Dr. Bright's notions, which he is here so anxious to disclaim; but we did believe him to suppose that some condition, whether functional or organic, connected with the disease he has described, is the sole cause of albuminous impregnation,—and that he does even still maintain this appears clearly from the passages just cited. We would point out, too, the curious fact that in the expression "the disease attending albuminous urine," Dr. Bright appears to make the latter the cause of the former.

In the majority of cases, abundant and persistent impregnation of the urine with albumen is indicative of the presence of Bright's disease; but Dr. Morrison's case, formerly referred to, even if no other of the kind had been met with, would suffice to prove that such abundance is not a pathognomonic sign. There are few cases, however, in which, if to such impregnation be joined deficiency of saline constituents and low specific gravity, the observer may not with confidence diagnosticate Bright's disease. Dr. Christison, though in the following brief summary showing how useful the characters of the urine may become in this respect, wisely cautions the practitioner against trusting to these alone, when he has it in his power to obtain additional information from other local and general signs, the secondary affections, &c. It will be perceived that in these propositions Dr. Christison again exhibits the peculiarity of his opinions respecting some points already referred to.

"1. When the disease has continued for a short time with acute symptoms, the characters of the urine—namely, a somewhat reduced density, a diminished amount of daily discharge of solids, and high coagulability—are invariable, and do not occur conjunctly so far as is yet known in any other disorder. 2. There is a very common conjunction of characters in the advanced stage, which has seemed to me never to occur in any other malady, namely, great reduction of density, some diminution of quantity, much diminution of the daily discharge of solids, and slight coagulability. 3. Another conjunction, not less characteristic perhaps, is great reduction of density, slight coagulability, and a great increase in quantity, consequently with little or no diminution of the daily discharge of solids. 4. I have never in any circumstance, except in the advanced stage of granular disorganization of the kidneys, met with urine about the natural standard in quantity, of the very low density of 1006 or 1008, consequently defective materially in the daily discharge of solids, almost colourless or cherry-red, or smoke-brown, or orange-yellow, and obscured by opalescent muddiness which does not disappear under rest or gentle heat,—even though not coagulable. 5. Though not absolutely prepared to state the same proposition where the quantity of urine is superabundant, and its other qualities such as those last described, I am inclined to think this condition also characteristic." (p. 56.)

The supervention of anasarca gives great additional certainty to the diagnosis: Dr. Christison supposes that the characters of this anasarca will in themselves sometimes suffice for its establishment. He has not for nine years (the number of cases observed would have been a better criterion of the value of the statement) met with a single case of inflammatory dropsy, where there were not unequivocal signs of the kidneys being diseased: he admits, however, that there may, as before intimated, be some doubt whether this is the fact in cases of dropsy consequent on scarlatina. A character frequently observed in the œdema depending on the renal disease is, that the part does not pit on pressure: Dr. Christison considers this peculiar to it. Again, "all dropsies where the urine is steadily above the healthy standard in point of quantity occur," according to this author, "in connexion with granular kidney, except in the instance of dropsy attending the advanced stage of saccharine diabetes." He is also inclined to think, though on this point not prepared to speak with confidence, "that all dropsies where the urine not being above the healthy standard in quantity, is also below 1010 in density, are connected with granular disorganization of the kidneys, whether the urine be albuminous or not."

In the acute stage of Bright's disease when the urine is bloody and albuminous, and dropsy has not yet appeared, the case might be mistaken for essential hæmaturia, or that occurring in hemorrhagic purpura, or vice versâ; but in the latter, blood is voided in large quantities and in clots; one of the renal regions is commonly more painful than the other, and the qualities of the urine usually change in the course of twenty-four hours. Of the presumed distinction respecting transmutation into albuminuria, we have already stated the inaccuracy. We can add little to our previous statement of the difficulties attending the distinction of the incipient stage of Bright's disease; if dropsy be present, the case is clearly of the latter description; if this be absent, the diagnosis seems, except under particular circumstances, to be in the present state of knowledge impossible. The passive renal hyperæmia attending diseases of the heart is oftentimes with difficulty distinguished from the renal disorganization; for although the albuminous urine of the former is generally of healthy specific gravity and contains the normal amount of solids, yet it is occasionally much less dense than in health. Under the latter circumstances M. Rayer confesses that it is impossible to determine whether the dropsy and albuminuria depend on the cardiac malady alone or on superadded renal disease. The œdema depending on the morbid states of the heart usually commences with the ancles, and even when advanced is diminished by the horizontal posture: that occurring during the disease of the kidneys frequently exhibits itself first about the face, and is little benefited by the recumbent posture. On the distinctive marks of encephaloid and tubercle of the kidney M. Rayer has not yet spoken.

A few words may here be appropriately introduced relative to the presumed frequency of the disease. Those who have written most enthusiastically on the subject seem to us to have exaggerated this most remarkably, or at least, beyond all question, to have drawn their inferences from incorrect data. Take for example Dr. Bright, who, in order to ascertain its frequency, "instituted a series of experiments by taking the patients promiscuously as they lay in the wards and trying the effects of heat upon the urine of each and at the same time employing occasionally other reagents." We need only refer the reader to the table given in our former article of the causes of albuminuria, and to what has been said in the foregoing pages, to convince him of the insufficiency of this sign as evidence of the presence of the disease, more especially when, as appears to have been the case with Dr. Bright's experiments, the specific gravity of the urine was not examined. Need we wonder that he found the proportion of persons labouring under the disease he has described "at least one in six, if not in four?" And here is the calculation referred to with commendation by Dr. Christison, (Pref., p. ix.)—by Dr. Christison who elsewhere rejects, as an unfair interpretation of his opinions, the notion that albuminuria is pathognomonic of the disease.

In a chapter entitled Pathogenesis, wherein M. Solon endeavours to trace the mode of formation of the morbid changes of the kidney, this observer states his belief that Bright's granulations are a product of interstitial exhalation and not a degeneration. Valentin's conclusion on this point, drawn from the microscopical examination of the kidneys of a child in which the cortical substance was of yellow colour and the external surface presented spots of ash-gray tint, is of a very different kind. This micrologist found the uriniferous ducts and the substance separating them perfectly normal, and ascertained that the vessels presented no unusual appearance either in respect of their diameter or distribution,—in fact, that the renal tissue possessed its healthy characters. The yellow discoloration appeared produced by a quantity of matter of that colour filling the ducts of the cortical substance, and composed of small granulated patches, of irregular and variable shape, and of yellow roundish globules. The tubuli of the tubular substance contained matter of the same kind, but in much less quantity. From these observations Valentin infers that the disease does not affect the tissue of the kidney, that this is in truth unchanged, and merely the receptacle of urine of modified properties: and hence that the albuminous state of the urine is in reality the cause of the deceptive appearance of disease in the renal tissue, instead of being the effect of real disorganization; he further infers that a morbid change of the blood leads to that of the urine. Whatever confidence the name of Valentin may warrant in the accuracy of this description, the reader will not forget that it is derived from a single observation of one form only of the disease, and therefore decidedly requires confirmation. Nor does this confirmation appear likely to be soon obtained—at least, the enquiries of Gluge seem to have led to the very different result, that the disease essentially consists in a deranged state of the capillary circulation of the cortical substance and especially of Malpighi's glands.

Is Bright's disease an inflammatory affection? In commenting formerly upon the term albuminous nephritis, employed by M. Rayer to designate the malady, we admitted the extreme probability that such was the fact, and the details of this author's present volume very distinctly show, as it appears to us, that such is the truth. "There is no disease," observes M. Rayer, "in the course of which the phlogistic diathesis is more distinctly marked, none wherein it manifests itself in a greater variety of situations or under a greater number of forms. ...... In the first stage of the disease, the kidney is red, swollen, and sometimes painful; and, if we are unable to ascertain whether it be hot, we are at least certain that the changes it has undergone are productive of fever." If the inutility of the antiphlogistic treatment during the advanced stages of the disease, and its want of influence over its chronic anatomical forms generally be urged in objection to this doctrine, we reply with M. Rayer, that such is the character of the consecutive lesions of all inflammatory affections. Will bleeding remove the intestinal ulcerations and indurations of chronic dysentery—the false membranes and puriform effusion of chronic pleurisy? And, because venesection is unavailing for this purpose, shall we expunge dysentery and pleuritis from the list of inflammations? Precisely the same line of argument may be adopted in answer to those who urge that the disease occasionally commences without active signs of inflammatory action. The qualificative "albuminous" cannot, in strictness, be reserved, as already stated, for this special form of inflammation, because simple nephritis is also attended with albuminuria.

With respect to the manner in which albuminous impregnation is produced, we need scarcely say that Valentin's notion is not that generally admitted, and that the majority of writers believe the renal disease to be the cause of that impregnation. But how are the two phenomena connected? If, as M. Solon observes, we suppose that the morbid condition of the tissues causes the serum of the blood to pass by simple transudation into the urinary passages, it may be objected that were this the case, the renal excretion should carry with it the other constituents of the blood also, which is by no means the fact. It contains no fibrine, and may hold colouring matter in suspension only temporarily, or in many cases not at all. Besides, if the serum of the blood escaped in the manner supposed, the liquid portion of the fluid remaining in the circulating system, should be proportionally less abundant than natural: now the very contrary is the truth—its relative quantity is greatly beyond the normal standard. M. Solon considers it more probable that the morbid state of the urine results from imperfect elaboration of the materials upon which the kidney acts in the normal state; in confirmation of this view, he cites the presumed fact, that the more complete the disorganization of the kidneys, the greater the quantity of albumen discharged with the urine; but, as already mentioned, the accuracy of this fact is altogether denied by Dr. Christison. The subject is plainly still open to enquiry.

Again, assuming as a position, which will be readily granted, that the anasarca is in direct dependence on the morbid state of the kidney, it may be enquired what is the mode of connexion between them. Sabatier and Martin-Solon seem inclined to believe that it depends on the increased proportion of serum in the blood and on its greater tenuity, the experiments of Magendie having proved that the facility of exhibition increases with the tenuity of fluids. Dr. Christison fancies that his experiments on the constitution of the blood lend force to this theory. It is, however, completely overturned by two facts thus stated by M. Rayer: "We know for a certainty, on the one hand, that patients affected with albuminous nephritis after scarlatina rapidly become dropsical, before any considerable quantity of albumen has been discharged with the urine; and, on the other, that individuals labouring under the endemic hæmaturia of tropical climates void albumen for years, sometimes during their entire lifetime, without becoming dropsical." (p. 608.)

Dr. Christison devotes twelve pages to the consideration of the prognosis of Bright's disease, examining successively the chance of recovery from the fundamental malady, the probability of relief from the secondary affections, and the particular symptoms which indicate approaching amendment or the contrary. With respect to the first of these points, cases of acute and chronic character, must be considered separately. The former appear to be as certainly curable, as the latter, so far as present knowledge permits us to affirm, is beyond the reach of art. The chronic forms of the disease are, sooner or later, in truth, almost invariably fatal,—but the fatal event may be averted to—we are almost justified in saying—an indefinite period, provided anasarca have been completely removed and the patient carefully avoid all excess of every description and rigorously live according to hygienic rule. With these precautions, a life of very tolerable comfort, with freedom from every direct effect of the disease except albuminuria, may be generally ensured. Respecting the secondary disorders, it is sufficient to say in general terms that they are distinguished for obstinacy with the exception perhaps of dropsy, which is generally observed to diminish considerably or disappear in patients of the lower orders who exchange the slavery, privation, and disorder of their ordinary life for the ease, comparative luxury, and regularity of an hospital: to this change, at least, may be attributed much of the good effect produced, much more than the criers-up of the various and often opposing modes of treatment under which it has been observed to occur, are likely to be willing to admit. As to the special symptoms announcing amendment, or the contrary, we must be prepared, from what has already been said on the differences of opinion regarding the relation of the modifications of the urine to the stages of the disease, for want of unanimity between the authors. Dr. Christison, of course, considers "the risk to life by no means proportioned to the amount of albumen in the urine, and that the reverse holds true in some measure." Rayer, Martin-Solon, and all those who maintain that the quantity of albumen increases with the advance of disorganization, must of course hold an opposite persuasion. And it is remarkable, that M. Rayer seems to think that the fact of the urine containing a small proportion of albumen would place the patient "in perfect security," were it not that, under such circumstances, death has been known to occur suddenly from intercurrent affections of the lungs or brain. Dr. Christison, however, qualifies the above statement very considerably in the following observations: "If attended with a moderately high or gradually increasing density of the urine, whether with or without an increase in its quantity, the diminution and disappearance of albumen are favorable signs. But this will, on the contrary, rather indicate a gradual advancement of the disease, if the density of the urine should at the same time slowly decrease, especially where its quantity remains stationary. Diminution of the albumen, with increased quantity and diminished density, cannot be relied on as a prognostic on either side." On the whole, the patient's danger would appear to be in proportion to the lowness of the density of the urine, unless the quantity of daily discharge is considerably above the standard.

Respecting the treatment of the disease in the acute stage, authors are highly unanimous. Antiphlogistic remedies of active character are recommended by all,—and of course, the most important of these, bloodletting. "When there is no contra-indicating circumstance from age or constitutional infirmity, whether original or acquired, the extent to which bleeding is carried should," according to Dr. Christison, "be regulated, as to extent and repetition, by the same rules which govern its employment in ordinary inflammations." This writer gives a hint of no mean utility, in enjoining careful examination of the blood drawn, more especially in respect of its colouring matter; by the proportion of the latter, he conceives, the degree of advancement of the structural changes in the kidneys may generally be ascertained; and if it should appear from the low proportion of colouring matter that the disease is not in its early but in some one of its more advanced stages, and hence the acute symptoms are not strictly primary, depletory measures should be pursued with much greater caution than under the contrary circumstances. The invariable accuracy of this mode of ascertaining the amount of progress of the disease may be doubted. Local abstraction of blood by cupping or leeching the renal regions, and the application of large linseed-meal poultices, are recommended by Drs. Bright, Barlow, and Rayer. The warm bath—if used beside the patient's bed—is advised by the same practitioners.

The restoration and maintenance of the cutaneous perspiration is one of the most important points to be attended to in the treatment of this affection, according to the testimony of the majority of those who have written upon it. Hence the value of keeping the patient in an equable and tolerably elevated temperature : so important is this, that we find Dr. Bright, who confines his patients closely to bed during the entire duration even of a protracted treatment, ascribing increase of anasarca to the passage of the cool air, allowed to circulate through the wards, over the bed-clothes of the patients. The use of flannel clothing is indispensable. Diaphoretics are advised in this stage by the majority of writers, and after the employment of more active remedies are probably useful; but the best diaphoretic treatment is here, as in inflammatory affections generally, the antiphlogistic. Dr. Osborne's views respecting the administration of medicines of the class just referred to, have been too long before the profession to require discussion at our hands.

The notions of Dr. Barlow on this point, though briefly alluded to in one of Dr. Bright's early papers, having for the first time been fully explained in the last number of the Hospital Reports, may be here more fully alluded to. This physician, he informs us, struck with the slowness of recovery of patients treated by the ordinary plan of bleeding, &c., was induced to seek for some more effectual method of removing the disorder. Persuaded of the inflammatory nature of the complaint, he determined upon a trial of tartarized antimony; and the results have, he assures us, as usually happens with the devisers of novelties in therapeutics, fully answered his expectations;—and several cases, which at least prove that recovery will take place under the use of this medicine, are related. Some of these are valuable in a pathological point of view, as further contributing to prove the identity of the renal disease occurring after scarlatina with the affection described by Dr. Bright, and as exhibiting the tractableness of the complaint, when really occurring with the character of an acute disorder in adults. But the reader must not imagine that Dr. Barlow confines himself to the exhibition of tartarized antimony in diaphoretic doses—this appears a mere adjunct in the treatment, and the precise share of the sum of benefit obtained which is to be attributed to it, cannot very easily be established. Dr. Barlow "would not merely recommend tartar emetic in the acute form of this disease on account of its diaphoretic properties,—but on account of its power of lowering the heart's action, as well as its local effects upon the capillaries when it reaches them through the circulation." If the pulse be hard and full, the medicine may be given in nauseating doses,—if not, smaller doses are sufficient. Whatever share of converts Dr. Barlow shall eventually make to the propriety of his modification of the diaphoretic mode of treatment, it is certain that the latter is far from being held in general estimation. Rayer, whose expectations of benefit from its employment have been "rarely realized," has found that the effects of even the most powerful agent for producing perspiration—the simple or medicated vapour bath—have rarely been maintained beyond a few hours after each bath. Dr. Christison observes: "I have often resorted to the diaphoretic plan, sometimes with evident advantage, *much more generally* without success; and I must likewise add, that I have several times seen general perspiration, both spontaneous and from the use of diaphoretics, fail to produce any material relief." Yet, singularly enough, we are told in the next sentence "that no one can question the general propriety of the diaphoretic *method of cure*."

The advisability of diuretic medication in this complaint is likewise matter of dispute. Dr. Osborne thinks that, so far from being useful, it may actually produce the disease; Dr. Bright has little confidence in it; Dr. Christison, on the other hand, "has very seldom witnessed decided diminution of the dropsy unless where diuretics [diuresis] or purging was either artificially induced at the same time or arose spontaneously." This practitioner has particular confidence in digitalis and cream of tartar.

We should have felt desirous of examining briefly the peculiarities of treatment required in respect of the diseases termed secondary in this affection, but we find we have exhausted our space.

M. Rayer's volume terminates with an historical sketch, occupying one hundred pages, of the rise and progress of knowledge respecting the remarkable disease which has been under consideration—or, we should rather say, respecting its detached phenomena. In this most erudite dissertation, the laborious author traces the germ of our acquaintance with the subject in the very earliest writers: first, in regard of the connexion of dropsy and disease of the kidneys, secondly, of dropsy and coagulability of the urine. Nothing can be rendered more clear from this survey of the contributions of our progenitors than the great merit of Dr. Bright, and that this principally consists in his having detected the connexion between these *three* conditions.

Of the three treatises on Bright's disease, with the leading contents of which we trust we have now made the reader tolerably well acquainted, each has its separate merits and demerits. It is impossible, in justice, to withhold from M. Rayer the palm of superior philosophy and completeness in his mode of treating the subject; but, with all our conviction of the value of abundant detail, we are compelled to add that his volume sins by needless prolixity. M. Solon's treatise, without possessing any very distinctive excellence, is a useful and practical history of the disease; but contains numerous errors on points of minor importance. Dr. Christison's work is particularly valuable from the fulness of its statements respecting the condition of the fluids in subjects labouring under this affection; but its style is disfigured by a certain affectation which betrays its author occasionally into actual incorrectness. If we can understand the meaning of such phrases as "the habitudes of granular disease" (p. 89); "the familiar phlegmasiæ" (p. 134); "micturition was now reduced to once every hour" (p. 243); "structural derangement" (p. 134); "tubercular derangement of the liver" (p. 99); "kidney dropsy, &c.," their intelligibility is assuredly their only merit. Nor are Dr. Christison's notions on pathology generally, as here exhibited, always remarkable for soundness. He employs the word "degeneration," for instance, apparently without any precise idea of its signification, and writes of the "peculiar action of vessels which leads to granular deposition," as if this were not a thing utterly unknown. Elsewhere, Dr. Christison intimates his suspicion that "perhaps" percussion is a more delicate and precise method of ascertaining the state of the liver than by "feeling and handling the abdomen"—"unless where the enlargement is very considerable." We have, of late years, in common with our betters, been accustomed to consider this as the *only* satisfactory mode of ascertaining the dimensions of this organ; and to the neglect of it is to be attributed the frequent discovery during life of "greatly enlarged livers," which same livers are on dissection often found to have miraculously retreated behind the ribs! Nor can we, in justice to the student into whose hands this volume may fall, omit to notice the statement that "the pathological states of the solids are now so far at least known, that few enquirers will be tempted to take up this investigation with the strong hope of discovery." Now, we affirm that there is not a solid in the frame which will not furnish to him who undertakes the investigation of its morbid state on correct principles an abundant harvest of rich and valuable novelty. Is this an affirmation which admits of being disputed? What then, for example, we would ask, is known with precision of the morbid conditions of the brain in insanity? Who has yet taught us to distinguish, even with tolerable certainty, the diseased changes of this viscus unconnected with madness? Is it, or is it not, notorious that truly sound pathologists are often unwilling to hazard their reputation by making any diagnosis in cases of cerebral disease, and that more bold, and yet well skilled persons having announced a cerebral hemorrhage, sometimes find a tumour, or persuaded they have been treating a case of softening are mortified at the discovery after death of uncomplicated hemorrhage? Or to change the ground—even in respect of the organ with the diseases of which we are best acquainted—the lung—and of the disease of that organ which has been most thoroughly investigated—phthisis—much remains to be done;—let the reader examine the abstract given in our last volume of M. Fournet's doctrine of the anatomical proofs of the curability of tubercle in its *first* stage, and he will at once acknowledge the fact. Nay more, it is not long since M. Cruveilhier, no mean spokesman on such a subject, stated in one of his fasciculi, adducing at the same time proofs of his statement, that the anatomical characters of pneumonia are as yet only imperfectly made out. But the experienced reader may easily continue the catalogue for himself; we have thus far volunteered guidance in so simple a matter, for the sake of those only who have just risen from the benches of the schools.

